# The Press and the Nursing Council

**Published:** 1920-11-06

**Authors:** 


### The Press and the Nursing Council.

A VERY grave state of things has been revealed by the inspired communiqué recently published in the *Daily Herald*. Readers who have read our reports of the proceedings at the General Nursing Council will remember that at the meeting of the Council on September 25 the Press was requested at a certain point in the proceedings to refrain from making public the discussions in progress on the rules for registration. This request our reporter, of course, complied with, as did those of other papers. Unfortunately, the *Nursing Times*, through a most regrettable misunderstanding, ventured to make public part of the discussion and thus paved the way for refusal on the part of the Council to admit the Press on the next occasion during all but entirely formal business.

At the meeting of the Council on October 15 the subject of the "Hours of Employment Bill" was brought forward. The Chairman having stated that the proceedings to follow were private, asked if the Press present would give their word that they should be so treated. Mrs. Bedford Fenwick, however, protested against the presence of the Press.

We now find in the *Daily Herald* of October 27 a statement as follows:—"The *Daily Herald* is supplied from a reliable source with an interesting piece of information on the attitude adopted in regard to the working hours of nurses by the majority of the members of the General Nursing Council. . . . The Council at a meeting about a week ago, having excluded representatives of the Press, considered the Hours of Employment (No. 2) Bill, and it decided by thirteen votes to ten that nurses should be excluded from the forty-eight-hour week provisions. Among the minority of ten were the four representatives on the Council of the working nurses."

We are not concerned with the truth or falsehood of this statement. What we are concerned about is the fact that one of the Councillors has betrayed the secrets of the Council. Whoever the culprit may be, he or she has naïvely succeeded, by the not very subtle insinuation appended, in completely exonerating every member of the College of Nursing from the commission of this shameless breach of privilege.

